# Examining the Acceptability and Feasibility of the Compassionate Mindful Resilience (CMR) Programme in Adult Patients with Chronic Kidney Disease: The COSMIC Study Protocol

**DOI:** 10.3390/healthcare10081387

**Published:** 2022-07-25

**Authors:** Anna Wilson, Clare McKeaveney, Claire Carswell, Karen Atkinson, Stephanie Burton, Clare McVeigh, Lisa Graham-Wisener, Erika Jääskeläinen, William Johnston, Daniel O’Rourke, Joanne Reid, Sohem Rej, Ian Walsh, Michael McArdle, Helen Noble

**Affiliations:** 1 School of Nursing and Midwifery, Queen’s University Belfast, Belfast BT7 1NN, UK; anna.wilson@qub.ac.uk (A.W.); c.mckeaveney@qub.ac.uk (C.M.); c.carswell@qub.ac.uk (C.C.); sburton06@qub.ac.uk (S.B.); clare.mcveigh@qub.ac.uk (C.M.); l.graham-wisener@qub.ac.uk (L.G.-W.); j.reid@qub.ac.uk (J.R.); ikwalsh@doctors.org.uk (I.W.); mmcardle20@qub.ac.uk (M.M.); 2 MindfulnessUK, Tauton TA1 1SW, UK; karen.atkinson@mindfulnessuk.com; 3 Faculty of Medicine, University of Oulu, 90570 Oulu, Finland; erika.jaaskelainen@oulu.fi; 4 Northern Ireland Kidney Patients Association, Belfast BT9 7AB, UK; willjohnston1@hotmail.co.uk (W.J.); danielorourke1504@gmail.com (D.O.); 5 Department of Psychiatry, McGill University, Montreal, QC H3A OG4, Canada; soham.rej@mcgill.ca

**Keywords:** end-stage kidney disease, kidney transplant, mindfulness, compassion, resilience, quality of life, COVID-19 pandemic

## Abstract

Kidney disease is often progressive, and patients experience diminished health-related quality of life. In addition, the impact of the coronavirus (COVID-19) pandemic, and its associated restrictions, has brought many additional burdens. It is therefore essential that effective and affordable systems are explored to improve the psychological health of this group that can be delivered safely during the COVID-19 pandemic. The aim of this study is to support a new service development project in partnership with the UK’s leading patient support charity Kidney Care UK by implementing the four-session Compassionate Mindful Resilience (CMR) programme, developed by MindfulnessUK, and explore its effectiveness for patients with stage 4 or 5 chronic kidney disease or have received a kidney transplant. The study will utilise a quasi-experimental, pretest/posttest design to measure the effect of the CMR programme on anxiety, depression, self-compassion, the ability to be mindful, wellbeing, and resilience, using pre- and posttests, alongside a qualitative exploration to explore factors influencing the feasibility, acceptability, and suitability of the intervention, with patients (and the Mindfulness Teacher) and their commitment to practice. Outcomes from this study will include an evidence-based mindfulness and compassion programme for use with people with kidney disease, which is likely to have applicability across other chronic diseases.

## 1. Introduction

Patients living with end-stage kidney disease (ESKD), or who have received a kidney transplant, experience difficult physical and psychological symptoms. Depression affects up to one quarter of these patients [1] and the prevalence rate for anxiety, estimated between 12% to 52%, is much higher than the general population [2]. These symptoms result in reduced quality of life [1], suicidal ideation [3], and increased risk of nonadherence to haemodialysis [4]. Despite the burden caused by this psychological distress on patients, and the associated risks, it often remains undetected. Even if depression and anxiety are uncovered there is limited availability of specialist psychological or social support services. In addition, patients may not report concerns because of this deficit [5]. A recent report identifying the UK Renal Psychosocial Workforce [6] found inadequate provision of psychological and social work support with large variations and disparities across the country. Only 5% of 84 units employ the recommended number of psychologists (one whole time equivalent (WTE) psychologist per 1000 renal replacement therapy (RRT) patients in units with adequate renal social work and renal counselling support, or one WTE per 500 RRT patients in the absence of additional support), and 14% of units have no psychosocial staff dedicated to patients with kidney disease [6].

More recently, the coronavirus disease 2019 (COVID-19) outbreak has been designated a public health emergency of international concern [7], which has severely impacted the mental wellbeing of many people with kidney disease. The highest mortality rates have been in people over the age of 60 and those who are immunocompromised or have underlying health conditions [8]. Patients who have kidney disease, or who have received a kidney transplant, are at an increased risk of severe COVID-19 and, therefore, are advised to take extra precautions to reduce their risk of exposure to the virus [9]. This includes shielding, a form of strict social distancing that requires patients not to leave their home for any reason and make alternative arrangements for food and medications to be delivered [10], a difficult feat for patients typically attending multiple hospital appointments. Patients who undergo haemodialysis are unable to adhere to shielding guidelines, as they usually attend hospital three times a week for their treatment, increasing their risk of contracting COVID-19 and incurring psychological morbidity. Many patients already have diminished health-related quality of life [11], and the impact of COVID-19 brings additional burdens. It is therefore essential that effective and affordable systems are explored to improve the psychological health of this group [1], which can be delivered safely during the ongoing COVID-19 pandemic and beyond.

One such intervention is mindfulness, which has increased in popularity over recent years as the scientific evidence base grows [12]. Mindfulness is defined by Thich Nhat Hanh as “the energy of attention” [13]. It involves focused, purposeful, nonjudgmental attention on the present moment and embraces practices that include meditation. Mindfulness has long been used as a stress-reduction technique, is practiced globally, and is accessible, free, and inclusive [14]. The Mindfulness-Based Stress Reduction (MBSR) programme was developed at the University of Massachusetts’s Medical Center by Professor Jon Kabat-Zinn, as an 8-week course initially intended to complement ongoing treatment plans for patients living with a wide range of conditions and to support individuals’ personal wellbeing [15]. The MBSR has evolved into a programme that can be delivered in a wide range of settings such as education [16], workplaces [17], and prisons [18] as well as hospitals and healthcare settings. The programme focuses on training an individual’s attention on feelings and sensations within the body, and acceptance of these feelings and sensations without judgement [19]. Participants consciously focus their attention on both internal and external stimuli in order to remain in the present moment and reduce disruptive thoughts about the past or future. Benefits include reduced stress and anxiety levels, improved sleep, improved mood, and an increase in resilience and coping skills [20,21], all factors that contribute to improved mental health, wellbeing, and quality of life. In addition, people who meditate have been shown to have less age-related brain atrophy [22]. Unlike cognitive behavioural therapy, which is goal oriented, mindfulness meditation relies on nonjudgmental observation. By distancing themselves from difficult sensations and thoughts patients can achieve a greater acceptance of their condition [12].

Mindfulness has been used effectively to treat mental health conditions, including anxiety [23], depression [24], and addiction [25] most often via the eight-session MBSR programme. In addition, it has been incorporated into the treatment of chronic physical diseases such as cancer [26], inflammatory bowel disease [27], diabetes and coronary heart disease [28], and in pain management [29] to improve quality of life. Within nephrology, there have been relatively few studies examining the effects of mindfulness [30,31,32,33,34,35,36], but results from recent studies have been promising, with many patients reporting similar benefits to those with other chronic diseases. However, these studies have been limited by difficulties in the delivery of mindfulness sessions, including a lack of qualified practitioners, space for practical based mindfulness activities, noisy environments, cost considerations, and lack of standardised approach [30,31,32,33,34,35,36]. In one study, which took place in a haemodialysis unit, barriers to intervention delivery included frequent haemodialysis shift changes, staff or alarm interruptions, space constraints, fluctuating medical status of participants, and patient fatigue [31]. To overcome these limitations, effective and affordable alternatives are required to ensure that patients can experience the benefits of mindfulness and compassion which can support increased resilience [37]. In addition, with the onset of COVID-19, it is important that wherever possible, delivery is provided online.

This protocol presents a collaborative, new service development study, delivered as a partnership between Kidney Care UK (KCUK), Queen’s University Belfast, and MindfulnessUK, to pilot, including full evaluation, a new virtual mindfulness programme for patients with kidney disease. Presently, access to psychological and emotional support is not well provided or funded, which is one reason that KCUK provides a universal telephone counselling service. The charity is keen to explore the feasibility of delivering mental health support via virtual means as a cost-effective way to significantly reach and support more patients with mental health needs. While the study is not a formal economic evaluation of the intervention, costs incurred (e.g., Mindfulness Teacher, supervision sessions, PPI representation) will be shared in future publications.

An eight-session mindfulness programme is considered the gold-standard and shown to be most effective; however, recruitment and retention have been problematic as it is not always practical for people with ill-health or caring duties to attend [26]. Condensed programmes have excellent potential, but such bespoke courses require rigorous evaluation. MindfulnessUK has recently developed a new programme to help manage stress, develop resilience, and enhance wellbeing using evidence-based mindfulness and compassion skills and techniques, which can be delivered as a four-session course (the Compassionate Mindful Resilience programme) and is being offered nationally [38]. The study intends to trial this four-session programme with patients who have reached stage 4 and 5 CKD and patients who have received kidney transplants. The aim is to increase equitable access to evidence-based mindfulness practices with the potential to improve the lives of people impacted by kidney disease. The programme will be implemented, with the support of an Expert Advisory Group, and obtain preliminary data with standardised measures of anxiety, depression, self-compassion, mindfulness, wellbeing, and resilience in order to progress with a larger scale efficacy and effectiveness trial. We hypothesized that following the introduction of the CMR intervention, there would be an improvement in anxiety as measured by the Generalised Anxiety Disorder questionnaire (GAD-7) immediately postintervention.

### 1.1. Aim

The aim of this work is to support a new service development project with KCUK by implementing the Compassionate Mindful Resilience (CMR) programme developed by MindfulnessUK and explore its effectiveness for patients who have kidney disease.

### 1.2. Objectives

(1) To implement the Compassionate Mindful Resilience (CMR) programme with an interdisciplinary Advisory Group including representatives from MindfulnessUK and KCUK for use with people with kidney disease.
(2) To measure the effect of the CMR on anxiety, depression, self-compassion, mindfulness, wellbeing, and resilience using pre- and posttests.
(3) To explore factors influencing the feasibility, acceptability, and suitability of the intervention with patients (and the Mindfulness Teacher) and their commitment to practice.
(4) To develop strategies for sustainability of the programme once the study is completed via a Partnership Board including representatives from KCUK, MindfulnessUK, and Queen’s University Belfast.

## 2. Expert Advisory Group and Partnership Board

The Expert Advisory Group will include: all members of the research team - Dr Helen Noble, Nephrology Nurse and Academic; Professor Joanne Reid, Academic Professor of Nursing; Dr Claire Carswell, Research Fellow; Dr Clare McVeigh, Lecturer in Nursing; Ms Anna Wilson, Research Assistant; Dr Clare McKeaveney, Lecturer in Nursing; Mr Ian Walsh, Consultant Surgeon and Clinical Academic; Dr Lisa Graham-Weiser, Psychologist; Ms Karen Atkinson, Co-Founder MindfulnessUK; Dr Erika Jääskeläinen, Psychiatrist, Finland; Mr William Johnston, KCUK Advocacy Officer Patient; Mr Daniel O’Rourke, Transplant Recipient; and Dr Sohem Raj, Consultant Psychiatrist from the “Geri-PARTy” Lab at the McGill-Affiliated Jewish General Hospital, Canada. Dr Raj has completed mindfulness research with patients receiving haemodialysis and has an established relationship with the research team.

A Partnership Board will be established including representatives from KCUK (P Bristow, S Yianni, J Gough, and J Pilcher), MindfulnessUK (K Atkinson), and Queen’s University Belfast (H Noble). Others will be requested to join as indicated. This board will prioritise sustainability of a mindfulness intervention to ensure mindfulness is made more readily accessible to patients via KCUK once the study completes. This would feed into phase two of this study to commence once the present study is completed, which would see an expansion of mindfulness programmes to patients and carers.

## 3. Methods

### 3.1. Study Design

A quasi-experimental, pretest/posttest design will be used alongside a qualitative exploration of acceptability. The outcome of interest will be measured before and after participants receive the intervention, and qualitative interviews will be conducted with a sample of participants.

### 3.2. Participants

Kidney Care UK will promote the CMR programme with details of the study for up to 75 participants. The study will initially be advertised in the KCUK Facebook Support Group which has over 9000 members (approximately 70% female/30% male). The average number of visits to the Facebook page per month in 2020 was 86,505. If required the information can be sent to all 8500 individual subscribers to the KCUK email mailing lists (40% male, 58% female, 2% other; split: 95% patient or carer, 5% health care professional). Additionally, the study can be advertised via social media. Approximately 20,000 people follow KCUK’s social media channels (Facebook/Instagram/Twitter).

### 3.3. Preliminary Work

Prior to the study commencing, the research team will undertake preliminary work with the Expert Advisory Group in consultation with the Partnership Board to ensure that the preintervention assessment is appropriate for a kidney disease population. The Full Client Assessment, which is carried out before participants undertake the CMR programme, will be refined in consultation with members of the KCUK counselling service, MindfulnessUK and people living with kidney disease to ensure the assessment considers the unique needs of people with kidney disease. Appropriate adaptations will be made to the delivery of the CMR programme in consultation with representatives of the Advisory Group who are living with kidney disease, the Mindfulness Teacher, and Partnership Board to ensure it is suitable for people living with kidney disease. For example, patients may tire easily and may need more regular breaks than the general public. Dr Helen Noble, an experienced nephrology nurse and registered Mindfulness Teacher will deliver the first CMR programme, as agreed with KCUK, and the subsequent programmes will be delivered by the trained Mindfulness Teacher.

### 3.4. Participant Recruitment

People with kidney disease interested in participating in the CMR programme and associated study will be asked to complete an online form to register their interest and confirm that they understand and meet the inclusion and exclusion criteria for the study. Participants over 18 years, living in the UK and in stage 4 or 5 kidney disease or have received a kidney transplant, who are not currently undergoing psychotherapy or experiencing severe periods of anxiety, depression, mental illness, addiction, recent bereavement, or a traumatic life event and have the capacity to provide consent will be eligible for inclusion.

Potential participants will be sent an information sheet about the study and a consent form. The information sheet will explain that anonymity will be guaranteed. Participants will then be referred to the registered Mindfulness Teacher employed for the study, for a standardised formal assessment, which has been adapted in consultation with the Expert Advisory Group to suit the population, to be undertaken to assess their suitability for the CMR programme. Participants will then be sent the questionnaires and instructions on how to complete them before commencing the CMR programme. The Research Assistant will be available to help those requiring assistance to complete the forms. Each CMR programme will include up to 10 participants, with a total of 75 participants.

### 3.5. Procedure of Data Collection

The study will take place over 18 months. All participants in the CMR programme will complete measurements at four time points: a pretest measurement two weeks before the start of the CMR programme and a posttest measurement immediately post-CMR programme and at 3 and 6 months. Demographic information will be collected at the start of the study, which will include age, gender, ethnicity, socioeconomic status, relationship status, highest level of education, current occupation, chronic kidney disease (CKD) status, length of time since diagnosis and estimated glomerular filtration rate (eGFR), along with data from the participant assessment. Instruments will be sent to the participants by email or post and the Research Assistant will be available to assist where required.

### 3.6. The Intervention

The CMR course is designed to be delivered for two hours per week for four consecutive weeks, in a group setting, online. This can be adapted to fit with client training needs, but MindfulnessUK recommend that at least one week is allowed in between sessions to allow home practice to take place. The programme was developed by Karen Atkinson of MindfulnessUK, executive committee member for the British Association of Mindfulness Based Approaches, which upholds standards and builds integrity in the mindfulness field [39]. It was developed in response to a growing need for people to access mindfulness and compassion practices in an easy, accessible way and was adapted from the industry standard MBSR programme [15]. The CMR was developed to provide an introductory mindfulness course teaching simple, effective, evidence-based practices, skills, and techniques found in the MBSR programme. Like the MBSR, the CMR was developed to help people manage stress, but has a specific focus on enhancing self-compassion and resilience. Self-compassion is a core component of mindfulness, which provides individuals with improved emotional resilience and reduced self-judgement, and a source of strength and resilience when dealing with life stressors [40]. The CMR programme was developed from the understanding that supporting development of mindfulness, self-compassion, and resilience can help individuals in navigating their world.

The CMR creatively delivers evidence based long term and sustainable teaching of mindfulness and compassion to build resilience in individuals and throughout organisations. Courses are delivered via the Zoom online meeting platform. If a participant is unable to attend a session, they should inform the Research Assistant. The Mindfulness Teacher can record the teaching elements of the session to send to the participant who missed the session. No other participants will be recorded, and written consent will be obtained from all participants to ensure they are aware why the Mindfulness Teacher is recording these sections of the session.

#### 3.6.1. Who Is the CMR Course for?

The CMR can be delivered to any organisation/group of individuals/individual that teach, supports, and/or cares for others. No previous experience of mindfulness is necessary. This is an introductory course that teaches simple, effective, evidence-based practices, skills, and techniques to help manage stress, develop resilience and enhance wellbeing using mindfulness and compassion. The CMR programme offers a blend of learning through concepts, short practises, and exercises and will be delivered online.

#### 3.6.2. CMR Resources for Participants

(1) CMR Participants Resources Pack, either emailed or printed.
(2) Downloadable Mindfulness and Compassion Meditation Practices.
  - Compassionate body scan;
  - Compassionate mindful movement;
  - Affectionate Breathing.
(3) A PowerPoint is used for some of the course content but not distributed to participants.

To deliver the CMR programme, the Teacher must have one of the following educational backgrounds:

(1) Have gained registration with the British Association of Mindfulness-Based Approaches as a qualified Mindfulness Teacher.
(2) Have gained a Level 4 qualification ‘Integrating Mindfulness and Compassion in Professional Practice’.
(3) Have qualified as a Compassion Teacher through the Centre for Mindful Self-Compassion.

In addition, they must have completed the CMR training with MindfulnessUK. A member of the research team will be present in each of the CMR sessions to support the Mindfulness Teacher and to ensure participant safeguarding.

### 3.7. Managing Risk

No psychological risk is anticipated for participants. However, the research team are cognisant of the fact that it is important to reduce any potential risk of emotional distress. Engaging in the CMR programme will enable participants to place themselves in the present moment and focus on their thoughts, emotions, and feelings. This may cause participants to feel increased stress and anxiety if they are overwhelmed with their true emotions and feelings. In order to manage this, skilled and qualified Mindfulness Teachers will be employed who will be trained to assist participants to deal with difficult emotions that arise. Distress and disclosure protocols will be in place to ensure the safety of participants while taking part in the study.

### 3.8. Data Collection

#### Measurements

The outcomes of interest in the current study are the effect of the CMR programme on anxiety, depression, self-compassion, mindfulness, wellbeing, and resilience. The primary outcome is anxiety immediately postintervention.

(a) Anxiety: The GAD-7 is a brief seven-item self-report scale based on the Diagnostic and Statistical Manual of Mental Disorders-IV criteria for generalized anxiety disorder, with items scored from 0 (not at all) to 3 (nearly every day). It has excellent internal consistency (Cronbach’s α = 0.92) and test–retest reliability (intraclass correlation = 0.83) [41].
(b) Depression: The PHQ-9 is a brief nine-item self-report scale based on the Diagnostic and Statistical Manual of Mental Disorders-IV criteria for major depressive disorder, in which each item is scored from 0 (not at all) to 3 (nearly every day). Similar to the GAD-7, construct validity studies have demonstrated its internal consistency (Cronbach’s α = 0.89) and test–retest reliability (intraclass correlation = 0.87) to be excellent [42].
(c) The Self-Compassion Scale (SCS) is the most commonly used measure of self-compassion; however, concerns have been raised that the 26-item SCS is burdensome for some individuals [43]. The shortened 12-item Self-Compassion Scale-Short Form (SCS-SF) [44] will therefore be utilised in this study.
(d) The Five Facet Mindfulness Questionnaire (FFMQ) is a self-report measure that is based on a five-facet model (i.e., Observe, Describe, Act with Awareness, Nonjudge, and Nonreact). The test consists of 39 items that measure the five facets, and the scores provide an estimate of where we stand in terms of mindfulness and self-awareness [45]. Results show that the Italian FFMQ (which has a similar factor structure to the English version) has good to excellent internal consistency as a whole (alpha  =  0.86) with subscale consistency ranging from 0.65 to 0.81, and test–retest stability for the total score is 0.71 [46].
(e) Tennant et al. [47] modified the Warwick–Edinburgh Mental Well-Being Scale (WEMWBS) into the Short Warwick–Edinburgh Mental Well-Being Scale (SWEMWBS). The SWEMWBS consists of seven positively worded items related to mental wellbeing, focusing on psychological and eudemonic wellbeing. It has been translated, validated, and used globally to evaluate levels of wellbeing in different populations, and has been used as an outcome measure for evaluations of interventions aimed at improving mental wellbeing. Robust measurement properties alongside brevity makes SWEMWBS preferable to WEMWBS for monitoring mental wellbeing. The scale is easily understood, practical, and inexpensive.
(f) The Mental Toughness Questionnaire (MTQ48), a self-report measure of mental toughness, and an accompanying model comprising 4 core components, challenge, commitment, control, and confidence, with responses graded on a 5-point Likert-type scale anchored from 1 (strongly disagree) to 5 (strongly agree) [48]. It is the most widely used and researched resilience measure. The scale is currently being used in a number of mindfulness research studies at Queen’s University and Newcastle University. Whilst there remains debate regarding using MTQ48 in some areas, it can be viewed as the standard mental toughness measure to date with an overall test–retest coefficient of 0.90 [49,50].

### 3.9. Data Analysis

The Statistical Package for the Social Sciences (SPSS v.28) will be used to assist with data analysis. Recruitment, participation, and retention rate of participants will be recorded. Baseline demographic data including age, gender, ethnicity, socioeconomic status, relationship status, highest level of education, current occupation, CKD status, and eGFR will be collected to gain an exploratory understanding of the demographics of participants. Descriptive statistics will be used to present baseline demographic data. Categorical data will be presented as frequencies and percentages, while continuous data will be presented as means and standard deviations.

A repeated measures t-test will be conducted to compare the mean scores of the outcome measures before and after receiving the intervention to inform future evaluation of efficacy. A repeated measures ANOVA will be used to compare the difference in mean scores across the four data collection time points (pre, post, 3 months post and 6 months postintervention). A range of graphs and tables will also be used to present study findings.

### 3.10. Sample Size

A sample size calculation was conducted using G*Power software to determine the size of the sample necessary to detect a minimal clinically important difference [51] in the Generalised Anxiety Disorder questionnaire (GAD-7) [41]. The calculation was based on data from a study evaluating a complex intervention for the management of anxiety and depression in a chronic disease population [52]. A sample size of 75 will have 95% power to detect a difference in the primary outcome measure of 3 [52], assuming the common standard deviation is 6 [52], using a paired t-test with a 0.05 two-sided significance level, whilst also allowing for a 20% attrition rate. The aim is to recruit 75 participants to the study to account for a higher rate of attrition expected of the population and intervention [53,54]. Based on this calculation, the sample size of 75 is expected to be sufficient to detect a difference minimal clinically important difference across the outcome measures.

## 4. Qualitative Evaluation

The study will utilise a mixed-methods approach, in the form of a sequential explanatory design, which is characterized by the collection and analysis of quantitative data followed by a qualitative descriptive investigation [55]. The purpose of the qualitative investigation is to further explore the quantitative results in more detail, in particular how these relate to factors affecting the acceptability and suitability of the intervention with patients and the Mindfulness Teacher. The quantitative and qualitative results will be integrated when considering the outcomes of the study and proposing strategies for future sustainability of the programme [56]. The interview schedule will be guided by the RE-AIM framework, developed in 1999 in response to a need to have a framework to evaluate the potential for, or actual, public health and population impact [57]. The RE-AIM QuEST framework proposes open ended qualitative questions across the five RE-AIM dimensions of Reach, Effectiveness, Adoption, Implementation and Maintenance to guide evaluation [58].

Participants will be asked to maintain a diary to record home practice during the study. This is optional for the participant’s own record, may be referred to in the qualitative interviews, but will not be used in the data collection for the study.

### 4.1. Participant Recruitment

Patients and the Mindfulness Teacher will be offered the opportunity to participate in the evaluation. When a participant completes the CMR programme, they will be asked by the Research Assistant if they would be interested in taking part in a qualitative interview. If they are interested, they will be sent a participant information sheet containing contact details for the researcher. Participants will be given 48 h to consider participation, and a consent form will be provided for completion at the start of each interview. Participants will be recruited for the qualitative evaluation by purposive sampling and will include a range of participants considering gender, ethnicity, age, and stage of CKD. At least one participant from each CMR programme will be included in the interview process.

### 4.2. Data Collection and Management

There are no formal criteria for determining sample size in qualitative research, and as a result there are no rules to confirm if a sample is sufficiently small or large [59]. The participants will be purposively selected to participate in the study. A sample of approximately 20 patients will be recruited. According to Guest et al. [60], a sample of 12 participants is appropriate to saturate data. However, these are approximate sample sizes, and it is important to note that data collection will continue until no new information or issues are presented by participants and data saturation has been achieved.

### 4.3. Data Analysis

Semi-structured interviews will be recorded and transcribed verbatim and analysed thematically. Thematic analysis is a method of analysing qualitative data involving the searching for recurring ideas (called themes), within a dataset [61]. It is a “method of identifying, analysing and reporting pattern themes within the data” [61]. The interviews will be professionally transcribed. The data will then be stored electronically, allowing for coding and analysis. Data will be managed and coded using NVivo 11 qualitative data analysis software. Data analysis will follow a six-stage process: familiarization with data, generating initial codes, searching for themes, reviewing themes, defining and naming themes, and producing the reporting themes [62]. A sample of interviews will be coded by members of the research team led by the Chief investigator, Dr Noble, and the Research Assistant, all of whom have wide experience in qualitative research methodology, including thematic analysis.

## 5. Dissemination Strategy

Outcomes from this study will include an evidence-based mindfulness programme for use with people with kidney disease. It may also have applicability across other chronic diseases. Using a multi-faceted strategy that targets local, national, and international interdisciplinary audiences, findings and outcomes will be disseminated across clinical and tertiary healthcare sectors and through renal policy decision makers and patient organisations. This will occur through publications, conference presentations, and workshops. The protocol for the study, and the implementation and evaluation of the CMR programme will be submitted for publication in high quality peer reviewed journals and presented at national and international conferences including the British Renal Symposium and the EDTNA/ERCA annual conference. Service users will also be involved in the dissemination of findings, and researchers will collaborate with Patient Associations including the Northern Ireland Kidney Patient Association and KCUK throughout the study. A social media plan will be created to incorporate platforms such as Twitter and Facebook to disseminate information, progress, events, and findings from the study. The study will be promoted via the Renal Arts Group, which has an established website and an excellent social media presence. An annual progress report will be sent to the KCUK research committee. Any publications and conferences arising from the research will be communicated direct to the KCUK Secretariat. At the end of the study a clear lay summary of the study findings will be compiled to disseminate to patients, carers, healthcare staff, NHS, and HSC trusts and policy makers.

## 6. Conclusions

A full research-led evaluation of the CMR programme will not only provide additional psychological support to patients during the COVID-19 pandemic, but also assess whether this programme could be rolled out more widely to many more patients. This would supplement the in-depth and personal counselling service provided by KCUK and expand the charity’s direct patient support services in this core area of the charity’s work. While the benefits of mindfulness for patients with ESKD have been examined in previous studies [30,31,32,33,34,35,36], there is further scope for exploration of the feasibility, acceptability, and suitability of a condensed programme, which can be delivered online for this patient group. Additionally, the effect size estimates generated from this study have the potential to inform future definitive clinical trials in this area of research.

## Data Availability

Not applicable.
